# Long-term endogenous acetylcholine deficiency potentiates pulmonary inflammation in a murine model of elastase-induced emphysema

**DOI:** 10.1038/s41598-021-95211-3

**Published:** 2021-08-05

**Authors:** Rosana Banzato, Nathalia M. Pinheiro, Clarice R. Olivo, Fernanda R. Santana, Fernanda D. T. Q. S. Lopes, Luciana C. Caperuto, Niels O. Câmara, Milton A. Martins, Iolanda F. L. C. Tibério, Marco Antônio M. Prado, Vânia F. Prado, Carla M. Prado

**Affiliations:** 1 Departments of Medicine, School of Medicine, Universidade de São Paulo, São Paulo, Brazil; 2 Department of Bioscience, Federal University of São Paulo, Rua Silva Jardim, 136 - Vila Mathias, Santos, SP Brazil; 3 Department of Medicine Nephrology, Universidade Federal de São Paulo, São Paulo, Brazil; 4 Department of Biological Science, Universidade Federal de São Paulo, Diadema, Brazil; 5 Immunology, Universidade de São Paulo, São Paulo, Brazil; 6 Molecular Medicine Group, Robarts Research Institute, London, Canada; 7 Department of Physiology & Pharmacology and Department of Anatomy & Cell Biology, University of Western Ontario, London, Canada

**Keywords:** Respiration, Chronic inflammation

## Abstract

Acetylcholine (ACh), the neurotransmitter of the cholinergic system, regulates inflammation in several diseases including pulmonary diseases. ACh is also involved in a non-neuronal mechanism that modulates the innate immune response. Because inflammation and release of pro-inflammatory cytokines are involved in pulmonary emphysema, we hypothesized that vesicular acetylcholine transport protein (VAChT) deficiency, which leads to reduction in ACh release, can modulate lung inflammation in an experimental model of emphysema. Mice with genetical reduced expression of VAChT (VAChT KD^HOM^ 70%) and wild-type mice (WT) received nasal instillation of 50 uL of porcine pancreatic elastase (PPE) or saline on day 0. Twenty-eight days after, animals were evaluated. Elastase instilled VAChT KD^HOM^ mice presented an increase in macrophages, lymphocytes, and neutrophils in bronchoalveolar lavage fluid and MAC2-positive macrophages in lung tissue and peribronchovascular area that was comparable to that observed in WT mice. Conversely, elastase instilled VAChT KD^HOM^ mice showed significantly larger number of NF-κB-positive cells and isoprostane staining in the peribronchovascular area when compared to elastase-instilled WT-mice. Moreover, elastase-instilled VAChT-deficient mice showed increased MCP-1 levels in the lungs. Other cytokines, extracellular matrix remodeling, alveolar enlargement, and lung function were not worse in elastase-instilled VAChT deficiency than in elastase-instilled WT-controls. These data suggest that decreased VAChT expression may contribute to the pathogenesis of emphysema, at least in part, through NF-κB activation, MCP-1, and oxidative stress pathways. This study highlights novel pathways involved in lung inflammation that may contribute to the development of chronic obstrutive lung disease (COPD) in cholinergic deficient individuals such as Alzheimer’s disease patients.

## Introduction

Chronic obstructive pulmonary disease (COPD), a progressive, debilitant, and common lung disease is characterized by obstructed airflow from the lungs due to exposure to harmful gases such as cigarette smoke. Pulmonary emphysema, the main form of COPD, is caused by the rupture of the alveolar walls induced by the degradation of elastin^1^. Two accepted hypotheses regarding the development of emphysema are proposed: one related to the imbalance between pulmonary proteases and antiproteases that break down connective tissue, and the other, related to overproduction of reactive oxygen species (ROS)^2,3^. Both mechanisms induce pulmonary inflammation^4,5^, resulting in the influx of different cell types, including macrophages, neutrophils, T lymphocytes (mainly CD8+) to the airways, parenchyma, and pulmonary arteries^6,7^. Activation of inflammatory cells themselves does not directly induce alveolar destruction. They do not rupture the tissue; instead, they may release proteases, which weaken the septal walls, and mechanical forces then is able to rupture the walls. Moreover cytokines and ROS^4^ released by inflammatory cells can contribute to the maintenance of the vicious cycle. Pro-inflammatory cytokines are involved in the pathophysiology of COPD and they are increased in the epithelium^6,8–10^ and in bronchial alveolar lavage fluid (BAL fluid) of patients with COPD^11^. IL-6 has been was reported to be increased in exacerbated COPD^9,10^, and also in stable COPD patients^12^ MCP-1, a key chemokine involved in macrophage activation, has been shown to be upregulated in patients with COPD^13^ and is also involved in pulmonary inflammation and mucus hypersecretion^14,15^.

The cholinergic anti-inflammatory system is involved in the control of inflammation in several organs, including the lung^16^. The vagus nerve is the main constituent of the parasympathetic part of the autonomic nervous system and innervates the lung^17^. Acetylcholine (ACh), a classical neurotransmitter of parasympathetic fibers^18^, is synthesized in cholinergic nerve terminals and by many non-neuronal cells in various organs, such as spleen, heart, and lung epithelial^19^. Once synthesized, ACh is transported into vesicles by the vesicular acetylcholine transporter (VAChT)^20,21^. VAChT is essential for the release of ACh into the peripheral and central nervous system^21–23^ and changes in VAChT expression directly influence the release of ACh^23–26^. In airways, ACh can interact with muscarinic and nicotinic receptors^18,27–29^. Binding of ACh to muscarinic receptors found in airway smooth muscle cells^30^ triggers a potent bronchoconstriction in the lung^31^. To note, muscarinic receptor antagonists are widely used in the treatment of respiratory diseases^32^ Binding of ACh to nicotinic receptors in immune cells has an important role in the control of inflammation^19,33,34^ but the role of the cholinergic anti-inflammatory system in the lung is still little understood, particularly in COPD. Studies have suggested that the interaction of ACh with the nicotinic α-7 receptor induces inhibition of NF-κB translocation to the nucleus and, consequently, a reduction in the release of inflammatory cytokines^17,35^.

Because ACh has multiple biological and antagonistic actions in the lung, the consequences of cholinergic tonus decrease in vivo is still not fully understood. Decreased cholinergic tonus is involved in several pathologies such as Alzheimer, Dysautonomia, and others^35,36^. Our group has previously shown that VAChT reduction affects lung inflammation per se and predisposes the development of experimental asthma^37^ and lung inflammation induced by air pollution^38^. These results suggest that the cholinergic anti-inflammatory system is an important target to be explored in lung diseases. Thus, we hypothesized that long-term endogenous cholinergic reduction could affect the pathogenesis of emphysema. To test this hypothesis, pulmonary emphysema was induced in a genetically modified mouse model of cholinergic dysfunction [VAChT knockdown, homozygous (VAChT KD^HOM^)]^21^. VAChT-mutant mice exhibit approximately 65–70% reduction in VAChT levels, and show similar decreased levels of ACh release^21,23^. Here, we demonstrate that VAChT deficiency can exacerbate lung inflammation induced by elastase.

## Materials and methods

### Ethics statement

The animals were kept in environments with controlled temperature (21 to 23 °C), humidity and with a 12 h light/dark cycle, with access to water and food ad libitum, following the ethical principles of guidelines of the National Council of Animal Experimentation that regulates animal research according to Brazilian Federal Law and of “Principles of Laboratory Animal Care” formulated by the National Society for Medical Research. All the experiments described in this study were approved by the Ethics Committee for Research of the Hospital das Clínicas—Faculty of Medicine of the University of São Paulo (document number 0766/08). This study was carried out in compliance with the ARRIVE guidelines.

### Animals and experimental design

KD VAChT mutant mice were generated by targeting the 5 'untranslated region of the VAChT gene by homologous recombination as previously described^21,22^. A reduction of 65–70% of ACh release was observed at the neuromuscular junction of these mice^39^. Heterozygous mice were intercrossed to produce male KD homozygous VAChT (KD) and wild-type controls (WT) (6–8 weeks old) used in these experiments. They were divided into a. homozygous mutant mice submitted to the elastase protocol (VAChT KD^HOM^-PPE), b. wild-type mice submitted to the elastase protocol (WT-PPE); W. homozygous mutant mice submitted to the saline protocol (VAChT KD^HOM^-SAL); and d. wild type mice submitted to saline protocol (WT-SAL). A n of seven animals per group was performed.

### Induction of emphysema

To induce emphysema, animals were anesthetized with xylazine (5 mg/kg) (Rompun, Bayer, Sao Paulo, Brazil) and ketamine (40 mg/kg) (Agener Uniao, Sao Paulo, Brazil) and received instillation of 50 μL of porcine pancreatic elastase (PPE) solution (7 mg/mL and 6.6 units/mg, pancreatic porcine elastase type I/E-1250, Type I, Sigma Aldrich, St. Louis, USA) (0.677 IU) via an intranasal drop^40,41^. Control group received saline nasal instillation. All animals were evaluated after 28 days of elastase or saline treatment.

### Evaluation of pulmonary mechanics

Animals were weighed, anesthetized by intraperitoneal injection of thiopental (70 mg/kg) (Cristalia, Sao Paulo, Brazil), tracheostomized, and then connected to a ventilator for small animals (Flexivent, Scireq, Montreal, CA) at a tidal volume of 10 mL/kg, 150 breaths/min and a physiological positive end-expiratory pressure—(PEEP) of 3–5 cmH_2_O. Experimental data from the forced oscillation technique were obtained only after the animals were paralyzed with pancuronium bromide (0.2 mg/kg) (NovaFarma, Anapolis, Brazil). Based on a previously described model^41,42^ respiratory mechanics was characterized by tissue elastance (Htis).

### Bronchoalveolar lavage fluid (BAL fluid)

At the end of the mechanical evaluation, animals were exsanguinated through dissection of the abdominal aorta and the BAL fluid was collected. The trachea was cannulated and the BAL fluid obtained by washing the last of the airways with 3 × 0.5 mL of sterile saline solution^41^. For total and differential cell counts, the BAL fluid was centrifuged at 112.03×*g* for 10 min and the cell pellet was resuspended in 0.2 mL of sterile saline. The total number of viable cells was determined in a Neubauer hemocytometer counting chamber. Differential cell counts were performed on BAL fluid cytocentrifuge preparations (450 rpm for 6 min) (Cytospin, Cheshire, UK) stained with Diff-Quick (Biochemical Sciences Inc., Swedesboro, NJ). At least 300 cells were counted according to standard morphological criteria.

### Pulmonary morphometry

After collection of BAL fluid, the anterior chest wall was opened, the lungs were removed *en bloc* and fixed with 4% formaldehyde for 24 h under a constant pressure of 20 cmH_2_O. The lung was then transferred to 70% ethanol and subjected to conventional histological techniques.

### Alveolar diameter evaluated by mean linear intercept (Lm)

For conventional morphometry, an eyepiece with a coherent system of 50 lines, 100 points and a known area was attached to the ocular microscope. Lm, an alveolar diameter indicator, was evaluated by the point-counting technique^43^ in 20 non-overlapping lung parenchyma fields per animal with a × 200 magnification, as previously described^43–45^.

### Pulmonary remodeling

Histological sections were stained for collagen fibers using Sirius-Red (Direct Red 80, C.I. 35780, Aldrich, Milwaukee, USA) and for elastic fibers using Oxidate Weigert Resorcin-Fuchsin. Using the same ocular described above, we evaluated the volumetric ratio of collagen and elastic fibers in the alveolar tissue using a dot counting technique^46^. The volumetric proportion of collagen or elastic was determined by dividing the number of points that reach collagen or elastin by the total number of points that reach the alveolar septa. Measurements were performed at 10–15 lung fields for each animal at a magnification of 400 × and the results were expressed as percentage^44,45^.

### Immunohistochemical evaluation

Immunohistochemical staining was performed using anti-NF-κB antibody (1: 300, SC-109, Santa Cruz Biotechnology, Santa Cruz, CA), Mac-2 anti-mouse macrophages marker (1: 10,000, CL8942AP, clone M3 38, Cedarlane, ON, Canada), goat polyclonal anti-8-epi-PGF2α (1:500, IS-20, Oxford Biomedical Research, Oxford, England) and anti-MMP-12 antibody (1:6000, LS-C29 5305, LS Bio, USA) by the biotin–streptavidin–peroxidase method. 8-epi-PGF2α was used to evaluate oxidative stress since it is a characteristic of COPD physiopathology and this form is considered the predominant form generated during free radical attack of cell membrane^47^. For the negative control, the primary antibody was omitted from the procedure and BSA was used instead. Using the point-counting technique described above, we determined the density of positive cells expressing NF-κB, macrophages, and MMP-12 in the lung parenchyma and in the peribronchovascular area in 10–15 fields per animal. Measurements were performed at × 1000 on each slide^48^ and the results were expressed as positive cells/area (10^4^ μm^2^). The expression of 8-isoprostane was evaluated using a digital analysis system and specific software (Image Pro Plus v. 4.5 for Windows, Media Cybernetics, USA, https://www.mediacy.com/). Sections were stained with an 8-isoprostane antibody and captured using a microscope (DM2500, Leica, Wetzlar, Germany) attached to a camera (Leica), and images were fed into a computer using Qwin Plus (Leica) software (https://www.leica-microsystems.com/). The area stained with isoprostane (%) was expressed as the amount of isoprostane in a specific frame relative to the total tissue area within that frame and was analyzed in lung tissue and the peribronchovascular area.

All the morphometric analysis was performed by two researchers who were unaware of the study groups.

### Measures of cytokines

In 5 additional animals from each group described above, the lungs were removed and rapidly frozen to perform cytokine measurements on the lung homogenate. The Bradford protein assay (Bio-Rad Laboratories, Hercules, USA) was used to measure total protein as described^49^. A Milliplex mouse plex cytokine assay kit (Merck Millipore, Billerica, USA) was used to test samples for the presence of MCP-1 (monocyte chemoattractant protein-1), IL-6, IFN-γ, MIP-2 (macrophage inflammatory protein-2) and IL-10. The assay was read in the Bio-Plex suspension array system, and the data were analyzed using the Bio-Plex Manager version 4.0 software. Levels of the analyzed cytokines were obtained using standard curves ranged from 32,000 to 1.95 pg/mL, as previously described. Results of all cytokines were expressed as pg/mg of protein.

### Statistical analysis

Statistical analysis was performed using SigmaStat software (SPSS Inc., version 10, California, USA, https://systatsoftware.com/products/sigmaplot/). Normality was assessed by the Kolmogorov–Smirnov test and data were expressed as mean ± SEM. Parametric data were analyzed by two-way ANOVA (emphysema and VAChT deficiency), followed by the Holm-Sidak post hoc test. The level of significance was adjusted to 5%.

## Results

On day 28, animals were weighted and both VAChT KD^HOM^-SAL (23.74 ± 0.88) and VAChT KD^HOM^-PPE (23.51 ± 0.82) showed a reduction in body weight of about 13% when compared WT groups [WT-SAL: 27.46 ± 0.87; WT-PPE: 27.17 ± 0.88) (P < 0.001 and P < 0.01, respectively).

### Endogenous VAChT deficiency increases pulmonary inflammation in mice instilled with elastase but does not worsens the emphysema

**Figure 1:**
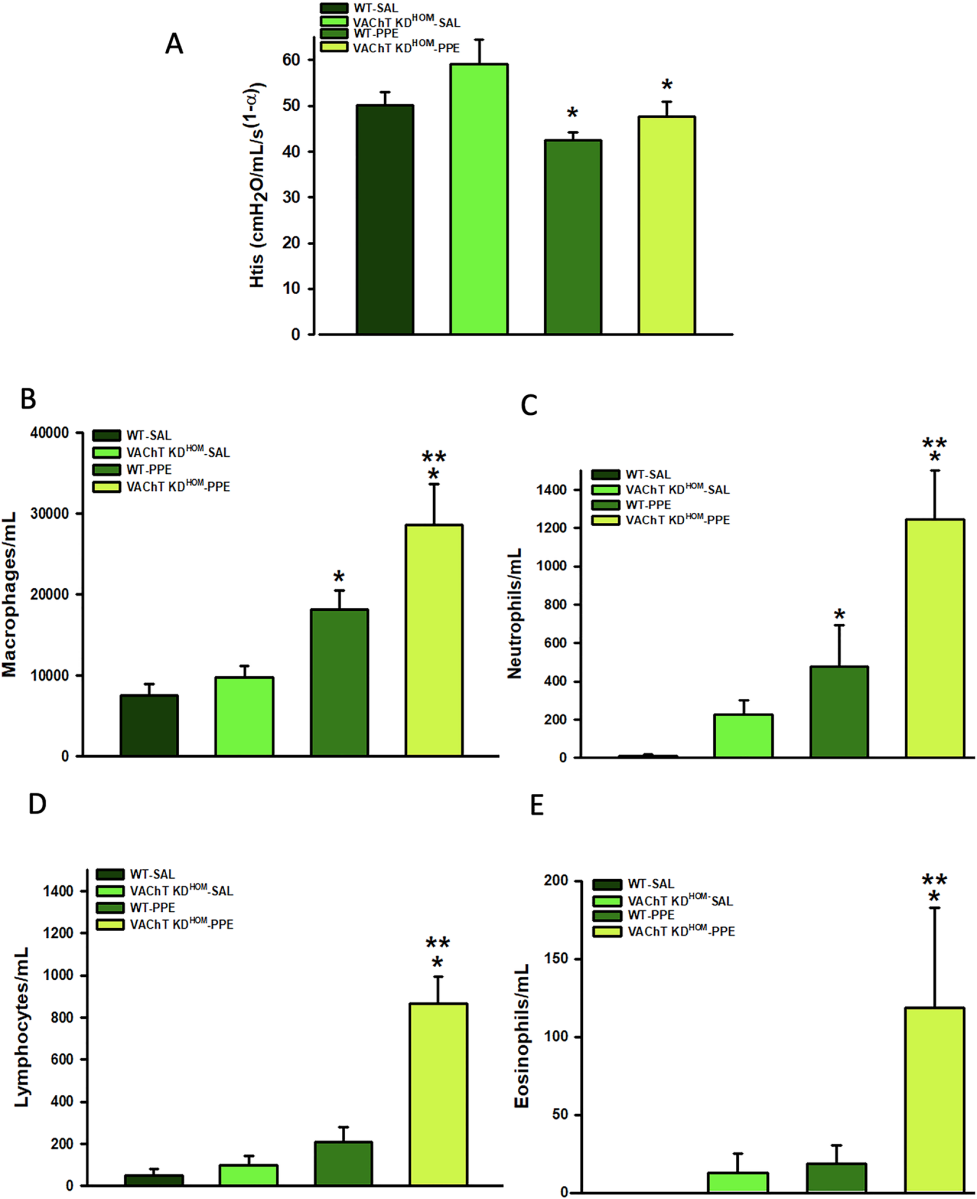
Respiratory mechanics and pulmonary inflammation. Data represent the mean ± S.E.M of five to six animals per group. (**A**) Tissue elastance-Hits. (**B**) macrophages, (**C**) neutrophils, (**D**) lymphocytes, and (**E**) eosinophils recovered from bronchoalveolar fluid (BAL fluid). *P < 0.01 compared to WT-SAL and VAChT KD^HOM-^SAL; **P < 0.05 compared to WT-PPE.

Both groups of mice submitted to the elastase protocol (WT-PPE and VAChT KD^HOM^-PPE) showed reduction in tissue elastance when compared to control animals that received saline (WT-SAL and VAChT KD^HOM^-SAL, P < 0.05 for both comparisons)], but the effect was similar in both genotypes, suggesting that cholinergic deficiency does not make tissue elastance worse (Fig. 1A). No significantly differences were found in airway resistance among experimental groups (data not shown).

We examined the role of VAChT deficiency in the susceptibility to elastase-induced lung inflammation in BAL fluid and lung. WT-PPE and VAChT KD^HOM^-PPE groups showed increased number of macrophages (Fig. 1B) and neutrophils (Fig. 1C) when compared to control mice that received saline (P < 0.05). Interestingly, while VAChT KD^HOM^-PPE mice showed increased number of lymphocytes (Fig. 1D) (P < 0.001) and eosinophils (Fig. 1E) (P < 0.05), there was no change in these parameters when WT-PPE were compared to WT-SAL.

**Figure 2:**
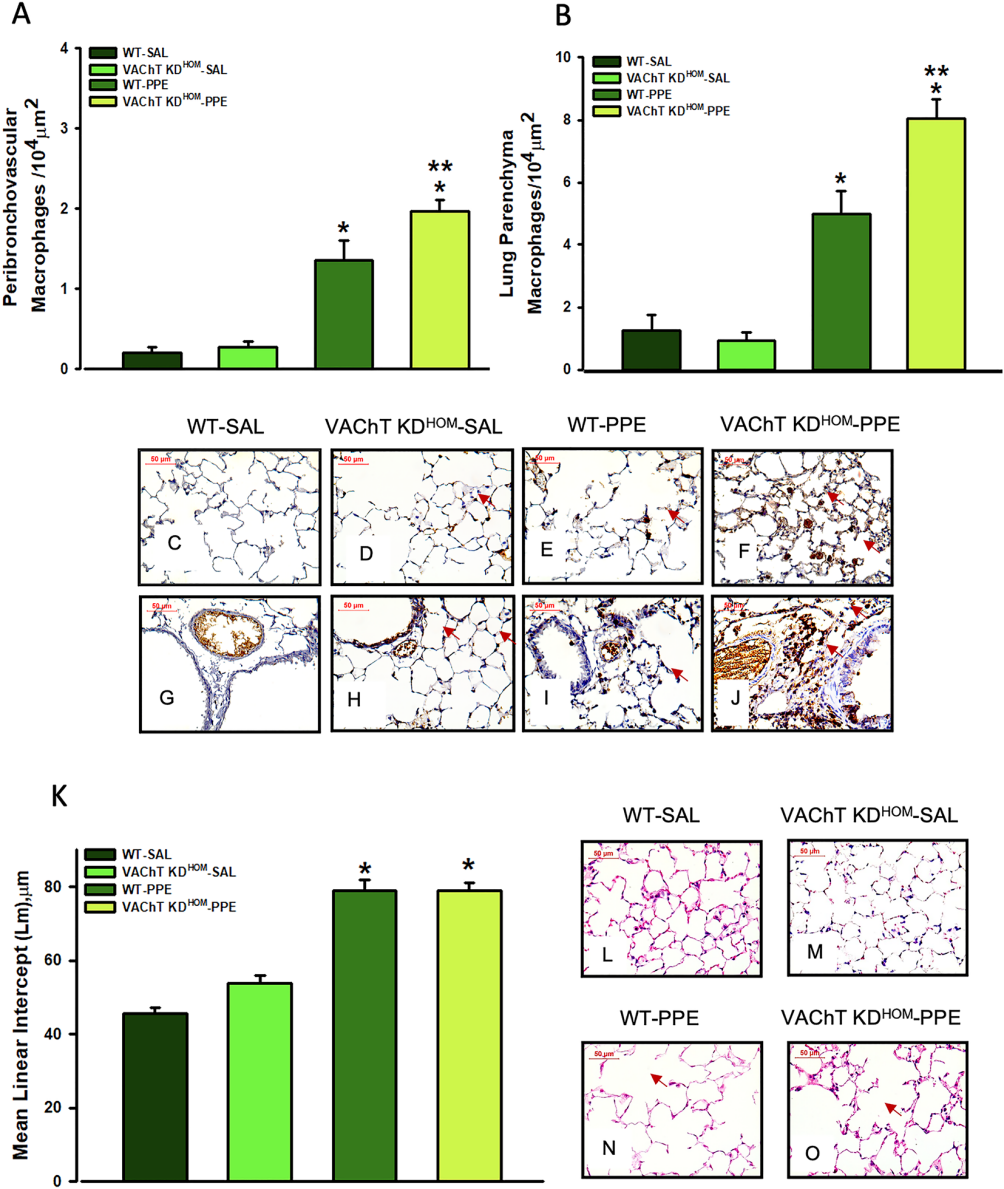
Macrophage MAC-positive cells and alveolar destruction (mean linear intercept, Lm). Data represent the mean ± S.E.M of six to seven animals per group. Macrophage MAC-positive cells in peribronchovascular (**A**) and lung parenchyma (**B**) and representative photomicrography from lung parenchyma and peribronchovascular area showing positive cells in animal lung from WT-SAL (**C** and **G**), VAChT KD^HOM-^ SAL (**D** and **H**), WT-PPE (**E** and **I**) and VAChT KD^HOM-^ PPE (**F** and **J**, arrows) groups. Data from alveolar destruction (**K**) and representative photomicrography (**L**–**O**, WT-SAL, VAChT KD^HOM-^ SAL, WT- PPE and VAChT KD^HOM-^ PPE, respectively. Arrows shows the alveolar destruction. *P < 0.001 compared to WT-SAL and VAChT KD^HOM^-SAL; **P < 0.05 compared to WT-PPE; ^#^P < 0.05 compared to VAChT KD^HOM^-SAL.

Both WT-PPE and VAChT KD^HOM^-PPE groups showed increased positive MAC2 cells, a marker of activated macrophages detected by immunohistochemistry, in peribronchovascular axis (Fig. 2A) and alveolar septa (Fig. 2B) when compared to saline group (P < 0.001 for all comparisons). As can be observed in representative photomicrographs (Fig. 2C–J), the elastase treated animals presented a strong positive stain in the macrophages (Fig. 2E,I). Also, macrophage infiltration in the alveolar septa and in the peribronchovascular area in VAChT KD^HOM^-PPE is clearly more marked (arrows) (Fig. 2F,J) than those observed in WT-PPE mice.

The Lm evaluation (Fig. 2K–O), a recognized parameter for detecting alveolar enlargement (emphysema), showed that WT-PPE and VAChT KD^HOM^-PPE groups have increased pulmonary airspace compared to control groups (WT-SAL and VAChT KD^HOM^-SAL, p < 0.001 for both comparisons). A comparative histological examination (Fig. 2L–O) clearly showed the presence of emphysema in the pulmonary parenchyma of animals with elastase (Fig. 2N). However, the effect was similar in both genotypes, suggesting that cholinergic deficiency does not worsens alveolar destruction (Fig. 2N,O).

To assess whether cholinergic deficiency interfered with cytokines release, we analyzed levels of IL-6, MIP-2, MCP-1, IL-10 and IFN-γ in the lung homogenate of the four groups studied (Table 1). All cytokines were similarly increased in WT-PPE and VAChT KD^HOM^-PPE mice when compared to saline groups (p < 0.05 for all comparisons), the only exception was MCP-1, which was more increased in VAChT KD^HOM^-PPE than in WT-PPE (approximately twofold increase).

**Table 1:** The effects of reduction in VAChT levels on pulmonary cytokines.

	WT-SAL	VAChT KD^HOM-^SAL	WT-PPE	VAChT KD^HOM-^PPE
IL-6 (pg/mg)	0.80 ± 0.18	0.97 ± 0.22	1.70 ± 0.18*	1.28 ± 0.22*
IL-10 (pg/mg)	5.09 ± 1.66	3.32 ± 2.14	12.29 ± 1.66*	9.97 ± 1.83*
IFN-ϒ (pg/mg)	1.73 ± 0.53	1.81 ± 0.68	3.62 ± 0.48*	4.45 ± 0.59*
MIP-2 (pg/mg)	14.20 ± 2.38	13.52 ± 2.92	21.55 ± 2.61*	20.70 ± 2.92*
MCP-1 (pg/mg)	12.7 ± 3.29	7.60 ± 4.25	17.27 ± 3.68*	32.34* ± 4.25*,**

Data represent the mean ± S.E.M of four to five animals per group. The levels of IL-6, IL-10, IFN-ϒ, MCP-1 and MIP-2 (pg/mg) in lung homogenate were increased in animals submitted to the elastase protocol compared to the saline groups. MCP-1 levels were increased in VAChT KD^HOM-^PPE group compared to WT-PPE.

*P < 0.05 compared to WT-SAL and VAChT KD^HOM-^SAL.

**P < 0.05 compared to WT-PPE.

### Endogenous VAChT deficiency increases NF-kB and 8-isoprostane PGF2α in peribronchovascular in mice instilled with elastase

**Figure 3:**
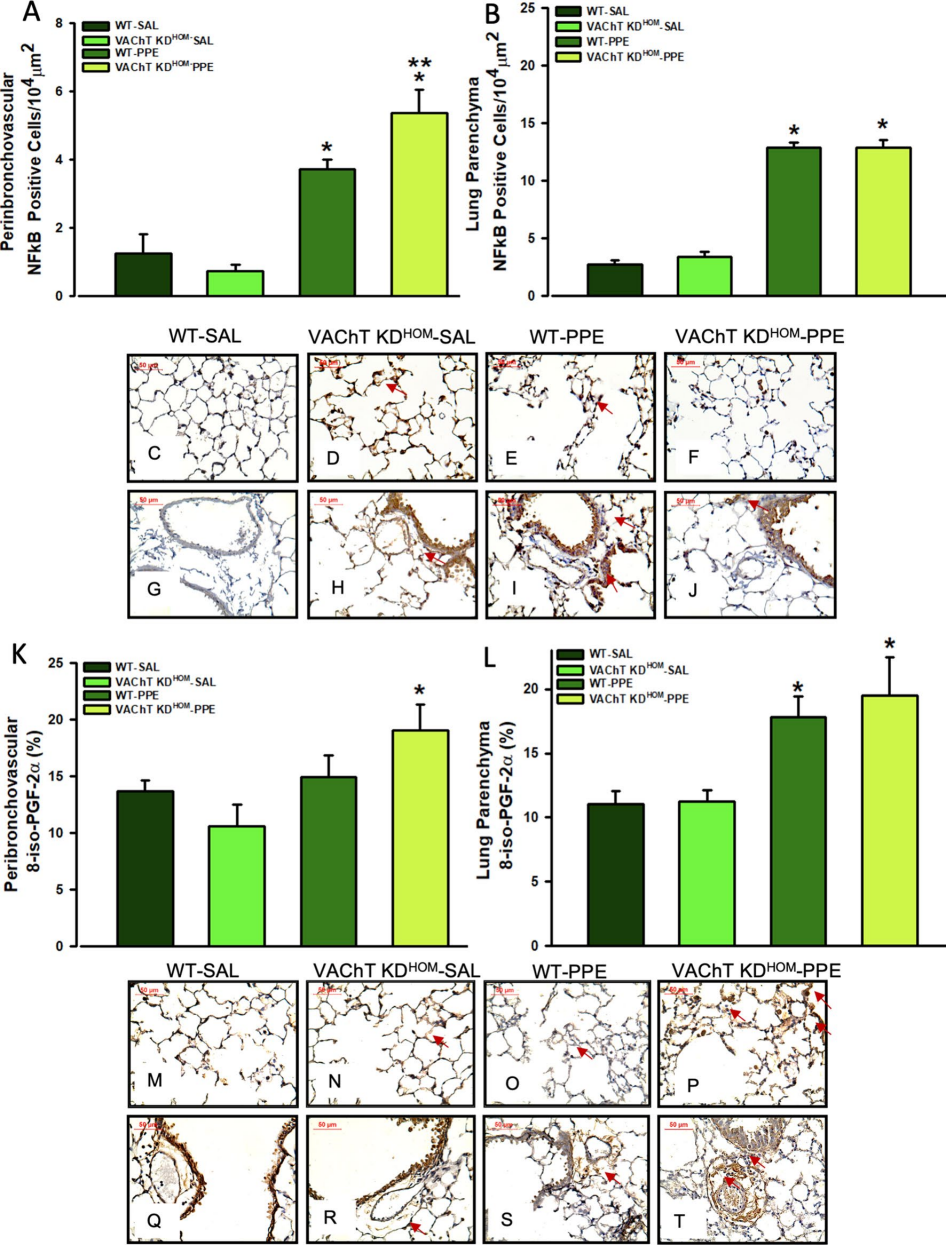
NF-κB positive cells and oxidative stress in peribronchovascular area and lung parenchyma. Data represent the mean ± S.E.M of six to seven animals per group. Number of positive cells to NF-κB in the peribronchovascular area (**A**), and in lung parenchyma (**B**) and representative photomicrography (**C**–**J**). The quantification of positive area for 8-iso-PGF-2α in the peribronchovascular area (**K**) and in lung parenchyma (**L**), and representative photomicrography (**M**–**T**). The NF-κB positive cells were detected by point counting technique and isoprostane positive area was detected using an image analysis system. Arrows point positive cells or area. **P < 0.05 compared to WT-PPE.

Activation of NF-κB is involved in the development of emphysema^50^. NF-κB positive cells were evaluated by immunohistochemistry, and we found an increase of NF-κB positive cells in both peribronchovascular axis (Fig. 3A) and lung parenchyma (Fig. 3B) in elastase treated control animals (WT-PPE) compared to saline groups (P ≤ 0.001). For VAChT-KD^HOM^-PPE mice, although in the lung parenchyma there were no effects on the NF-κB positive cells, there is increased NF-κB positive cells in peribronchovascular area compared to saline treated controls (P < 0.05). These findings are illustrated in the photomicrographs (Fig. 3C–H,J) where NF-kB-positive cells are represented by the arrows.

Isoprostane, a marker of oxidative stress^47^, was evaluated by immunohistochemistry. We found that WT-PPE and VAChT KD^HOM^-PPE mice showed increased 8-isoprostane-PGF-2α staining in the lung parenchyma (Fig. 3L) compared to saline groups (P < 0.05). VAChT KD^HOM^-PPE also showed an increase in 8-isoprostane-PGF-2α staining (P < 0.01) in peribronchovascular area compared to VAChT KD^HOM^-SAL (Fig. 3K), which was much stronger than that observed for WT-PPE mice. Representative photomicrographs showing slice of lung stained for 8-isoprostane are shown in Fig. 3M–T.

### Endogenous VAChT deficiency did not interfere with lung remodeling in mice instilled with elastase, although increased MMP-12 positive cells

**Figure 4:**
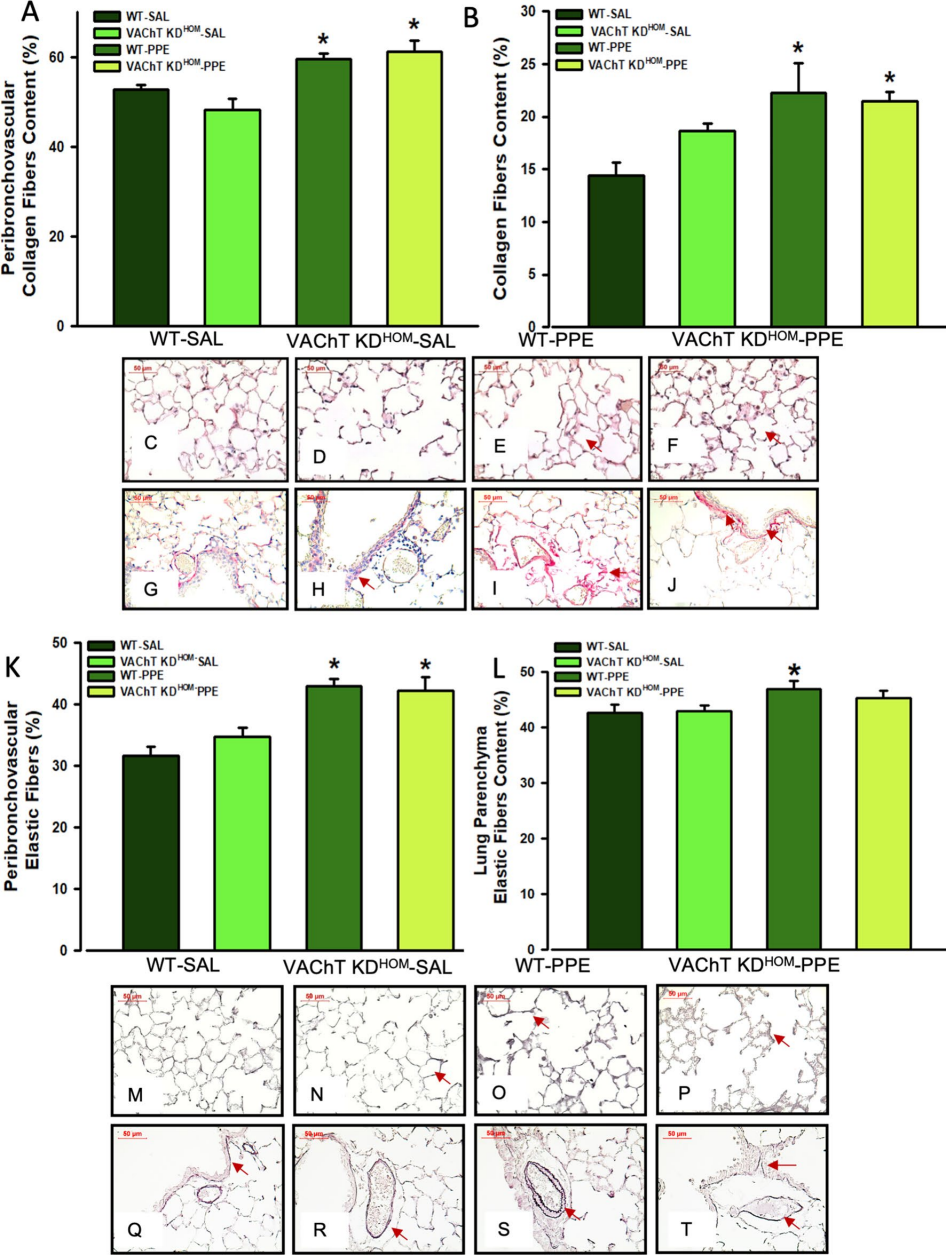
Lung parenchyma and peribronchovascular remodeling. Data represent the mean ± S.E.M of six to seven animals per group. Peribronchovascular collagen fibers (**A**), lung parenchyma collagen fibers (**B**), peribronchovascular elastic fibers (**K**) and lung parenchyma elastic fibers (**L**) were evaluated in paraffin sections stained with Picro-Sirius or Oxidative Resorcin Fuchsin, respectively. Arrows in panels (**C**) to (**J**) point the collagen fibers and in panels (**M**) to (**T**) elastic fibers in both lung parenchyma and peribronchovascular area. *P < 0.05 compared to WT-SAL and VAChT KD^HOM-^SAL.

Pulmonary remodeling is a characteristic of emphysema, and some features of remodeling are the deposition of extracellular matrix fibers. Both WT-PPE and VAChT KD^HOM^-PPE groups showed increase of collagen and elastic fibers deposition in both peribronchovascular tissues (Fig. 4A,G–K,Q–T) and pulmonary parenchyma (Fig. 4B–F,L–P) compared to saline groups (P < 0.001 for all comparisons). However, there was no significant difference between genotypes. These results suggest that cholinergic deficiency did not interfere in the deposition of extracellular matrix in the pulmonary parenchyma and in the peribronchovascular axis.

**Figure 5:**
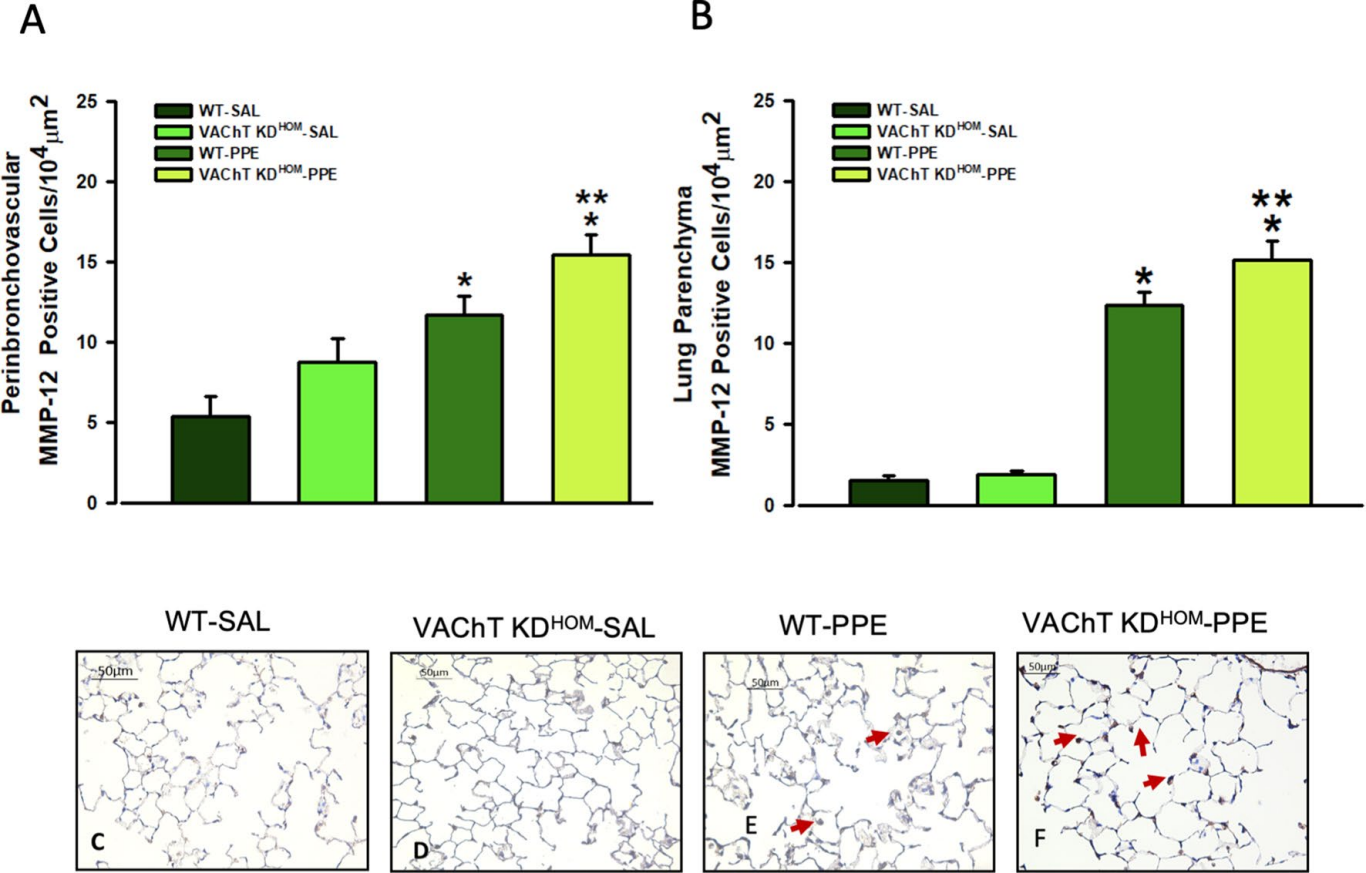
MMP-12 positive cells in peribronchovascular area and lung parenchyma. Data represent the mean ± S.E.M of six to seven animals per group. Number of positive cells to MMP-12 in the peribronchovascular area (**A**), and in lung parenchyma (**B**) and representative photomicrography (**C**–**F**). The MMP-12 positive cells were detected by point counting technique. Arrows point positive cells. *P < 0.01 compared to WT-SAL and VAChT KD^HOM-^SAL; **P < 0.05 compared to WT-PPE.

In Fig. 5, data from positive cells to MMP-12 in peribronchovascular area and lung parenchyma are shown. We found that WT-PPE and VAChT KD^HOM^-PPE showed increased number of positive cells to MMP-12 compared to respective saline groups (P < 0.01). However, in both pulmonary compartments, animals with cholinergic deficiency and emphysema (VAChT KD^HOM^-PPE) showed more augment of MMP-12 expression than observed in wild-type (WT-PPE) (P < 0.05).

## Discussion

The present study investigated whether long-term endogenous cholinergic deficiency is involved in emphysema development induced by elastase. The major finding in the present study is that VAChT deficiency increases pulmonary inflammatory responses induced by elastase, without affecting the emphysema development, lung function, and pulmonary remodeling. These results suggest that VAChT levels, and consequently ACh release, can modulate lung inflammation in an emphysema model reinforcing previous data that ACh has an important protective role against pulmonary inflammation in different models of pulmonary diseases^37,38^.

Emphysema is one of the COPD manifestations. Our data validated previous findings that a single dose of elastase instillation can induce a significant increase in alveolar diameter, and decrease tissue elastance, which are the main feature of emphysema^51^. Elastase instillation also induced an increase in macrophages and neutrophils in BAL fluid and an increase in MAC2 positive macrophages in lung tissue and peribronchiolar area. We also found increased levels of cytokines in the lung, as well as in oxidative stress and NF-κB positive cells in lung tissue and peribronchiolar area. Increased collagen and elastic fibers deposition in lung parenchyma and peribronchovascular area suggest a process of lung remodeling. Elastase instillation is not the most physiological way to induce emphysema, especially compared to human emphysema, which is most induced by cigarette smoke. However, this model show similar characteristics to the lungs of patients with COPD^1,6,14,52^ and has been used in other experimental studies of emphysema^44,45,53^.

Considering the multiple biological functions related to ACh and the importance of this mediator in inflammation, we hypothesized that changes in endogenous cholinergic neurotransmission affect the pathophysiology of emphysema. The administration of elastase in VAChT-KD^HOM^ mice worsened lung inflammation by increasing the number of macrophages, neutrophils, lymphocytes, and eosinophils obtained in BAL fluid. Cholinergic deficiency also induced an increase in the number of MAC-2 positive cells in the pulmonary parenchyma and in the peribronchovascular area. MAC-2 expression in macrophages has been shown to suggest that these cells were activated by inflammatory stimuli^44,45,53^. Macrophage plays an important role in COPD since these cells induce the release of several proteases involved in lung destruction and remodeling^7,54^. In addition, lymphocytes, especially CD8 + and neutrophils, are also involved in COPD, as they can release pro-inflammatory cytokines and proteases.

The bronchoconstriction action of ACh in muscarinic receptors has been intensely studied in lung diseases^30^. A role for ACh in nicotinic receptors has been recognized in acute models of inflammation. Nicotinic receptors are expressed in bronchial and alveolar epithelial cells, as well as in inflammatory cells, such as macrophagic cells, neutrophils, and lymphocytes^55–57^. Binding of ACh to α7 nicotinic receptors (nAChR) inhibits the production of TNF-α, MIP-2 and other inflammatory cytokines^19,28,34,58^. More related to COPD, Zhang et al.^59^ showed that the nAChR gene is a susceptibility variant for the development of COPD. In addition, Budulac et al.^60^ suggested that single nucleotide polymorphisms in the nAChR cluster are indirectly involved in the development of emphysema, interfering with smoking, increasing nicotinic dependence in humans. Recently, it was shown that the use of an agonist of nAChR7 suppressed the release of inflammatory mediators by human peripheral blood mononuclear (PBMCs) from COPD patients^61^.

We found that VAChT-mutant mice showed a two-fold increase in MCP-1, an inflammatory protein involved in the recruitment of macrophages. MCP-1 is upregulated in patients with COPD^13^ and is involved in mucus hypersecretion and influx of macrophages into the lung^14,15^. The major cell that produces MCP-1 is epithelial cells and macrophages and the last one is increased in VAChT animals that received PPE. In turn, macrophage infiltration is also regulated by MCP-1 release^62^. Therefore, we hypothesized that macrophages are the major source of MCP-1 in this model and the effects of VAChT deficiency in MCP-1 can be due to increase in macrophage. Also important, macrophage is the most important immune cell to express the nicotinic receptors involved in the anti-inflammatory effects of cholinergic system^17^. In addition, MCP-1 has been increased in mice with signal transducer and transcription activator (STAT3) deficiency^63^, a possible pathway involved in the anti-inflammatory cholinergic system^28^. In this regard, we previously showed that mutant mice to VAChT showed reduced expression of tyrosine kinase (JAK-2) in lung^64^, that probably inhibits STAT3 pathway, which can maybe explain the increased levels of MCP-1. Conversely, IL-6 and MIP-2, cytokines also involved in the recruitment of macrophages and neutrophils to the lung and has been found to be increased in the lung of patients with COPD^65,66^ were not differentially affected in VAChT-mutant mice.

The anti-inflammatory effect of ACh on α7nAChR is associated with inhibition of NF-κB translocation to the nucleus and consequent inhibition of cytokines released from macrophages and other cells^67,68^. NF-κB is involved in the pathophysiology of COPD and is also increased in the lungs of patients with COPD^69^. We found that elastase treatment in mutant mice induced an increase in NF-κB positive cells only in the peribronchovascular area compared to the WT-PPE group, suggesting that this signaling could be one of the mechanisms involved in the amplification of pulmonary inflammation observed in emphysematous and mutant mice. Interestingly that we found this effect in peribronchovascular area and not in lung parenchyma. This can be attributed to the fact that mice have a more pronounced inflammation in this region than in lung tissue or around airways different from what is observed in human lung with COPD^70,71^ or because the main source of ACh in lungs is the airway epithelial cells that can produce ACh by a neuronal and non-neuronal mechanism^27^. However, is important to note that in this study, the expression of NF-kB was evaluated in inflammatory cells that can be in this model, macrophages, lymphocytes, and neutrophils since they were detected in BALF.

Other mechanisms may also be involved in the anti-inflammatory effects of ACh such the effects of α7nAChR activation on the JAK2 and activation of the STAT3, thereby reducing the release of proinflammatory cytokines by the induction of SOCS3^63^.

Oxidative stress plays an important role in the development of COPD and induces deleterious effects on the respiratory tract of patients with COPD^72,73^. Instillation of elastase in VAChT-mutant mice induced an increase of isoprostane-8 staining in the peribronchovascular area that was not observed in wild-type animals submitted to the same protocol. These data suggest that increased oxidative stress may be a pathway that partially explains the data obtained in animals with VAChT reduction. Noteworthy, Roy et al.^74^ demonstrated that mice with VAChT deficiency in cardiomyocytes show increased oxidative stress in the heart.

Tissue remodeling can be defined by changes in the amount, composition, and organization of the extracellular matrix structure and it is a common feature of repair of tissue damage observed in COPD^75^. Interesting, that there is more alveolar destruction in VAChT-KD-SAL compared to WT-SAL, although both saline groups have less alveolar destruction compared to animals that received elastase. One possibility to explained it is the long-term deficiency of cholinergic tone which induces, per se, an inflammatory *milleau* as previously showed by Pinheiro et al. ^64^, although in the present study we did not found statically difference in lung inflammation between WT-SAL and VAChT-KD-SAL.

As cholinergic deficiency aggravates pulmonary inflammation, we expected that it would also affect pulmonary remodeling. However, morphological analysis of the lung revealed that long-term cholinergic deficiency did not affect the destruction and remodeling of the parenchyma in this model of emphysema. Our data also showed that tissue elastance changes induced by instillation of elastase is not worse in VAChT deficiency than in WT control mice. It makes sense since changes in extracellular pulmonary fibers are one of the most important determinants of pulmonary compliance changes observed in emphysema^75^.

Several evidence suggest that remodeling appears in response to inflammation and lung injury, however the cause-effect of inflammation, remodeling, alveolar destruction and lung function is controversial^76^. Ito et al.^77^ demonstrated that lung function and abnormal compliance observed in a mouse emphysema model were associated with collagen remodeling. In this case we did not found any alteration in collagen deposition or lung function between mutant mice and wild-type with emphysema. Another study that investigated pulmonary alterations in a papain-induced emphysema model^40^ observed increased number of macrophages starting one day after papain instillation while alveolar destruction, remodeling, and changes in elastance were evident only after day 15. The morphological changes were suggested to be more related to increased MMP-12 expression than to inflammation. In this regard we evaluated MMP-12 positive cells in both peribronchovascular area and in lung tissue and contradicts our hypothesis, cholinergic deficiency in emphysematous mice induced an increase in positive cells for MMP-12.

MMP-12 activity is associated to the destruction of alveolar walls and the use of MMP inhibitors in emphysema have been suggested^78^, however, Manoury et al.^79^ showed that mice deficient to MMP-12 did not improve the lung fibrose induced by bleomycin. One limitation of the present study is that we did not evaluate the MMP activity, and we looked to one point during the emphysema development (28 days). Therefore, the specific relationship between MMP-12, collagen deposition, alveolar destruction, and lung inflammation in VAChT emphysematous mice was not totally clear and maybe a time-course study will be necessary to better understand these findings. Together, our data indicates that the increase in inflammatory response was not the main determinant of lung function or alveolar destruction in this model.

In conclusion, we have shown that reduction of cholinergic signaling increases lung inflammation in a model of emphysema at least in the elastase instillation models, ACh exerts a protective anti-inflammatory effect without affecting emphysema and tissue remodeling. To the best of our knowledge, this is the first time that the role of VAChT in pulmonary inflammation in a model of emphysema has been reported. Although these data reveal a new pathway involved in the pathophysiology of COPD, further studies investigating how neuronal or non-neuronal cholinergic signaling contribute to the increase of pulmonary inflammation in emphysema are warranted. Endogenous cholinergic dysfunction in the long term, a situation commonly observed in several diseases, including heart failure, dysautonomia and Alzheimer’s disease^36^ may facilitate the development of COPD.
